# Complex psychiatric comorbidities in Thai trans women: A case series

**DOI:** 10.12688/f1000research.75144.1

**Published:** 2022-01-21

**Authors:** Sorawit Wainipitapong, Soravit Burakitpachai, Thanapob Bumphenkiatikul

**Affiliations:** 1 Department of Psychiatry, Faculty of Medicine, Chulalongkorn University and King Chulalongkorn Memorial Hospital, the Thai Red Cross Society, Pathumwan, Bangkok, 10330, Thailand; 2 Center of Excellence in Transgender Health (CETH), Faculty of Medicine, Chulalongkorn University, Pathumwan, Bangkok, 10330, Thailand; 3 Faculty of Medicine, Chulalongkorn University, Pathumwan, Bangkok, 10330, Thailand; 4 Division of Academic Affairs, Faculty of Medicine, Chulalongkorn University, Pathumwan, Bangkok, 10330, Thailand

**Keywords:** Transwomen, gender dysphoria, psychiatric disorder, mental illness, Case Study, Gender Dysphoria, Transgender, Mental Health, Gender Identity Disorder (GID), Trans

## Abstract

Trans women (TW) have a high prevalence of poor mental health. Gender-affirming treatments could reduce distress regarding their gender incongruity. However, psychiatric comorbidities might complicate the management or even confirmation of being transgender. We reported three TW with complex mental illnesses, including anxiety disorder with cultural explanation, neurodevelopmental disorders with cross-dressing, and severe personality disorder accompanied by major depression. All cases received both psychiatric and gender-affirming treatments, which demonstrated promising outcomes. Along with gender dysphoria (GD), psychiatric comorbidities also altered these TW’s identity and manifestations. Recognition of such conditions would be beneficial in providing care for all TW, both with and without GD.

## Introduction

Compared to the cisgender population, transwomen (TW) are at higher risk of developing mental disorders, including anxiety and affective disorders, suicidality, and substance abuse ( Schulman & Erickson-Schroth, 2019). Gender-affirming treatments could alleviate their distress but, even following gender transition, TW remain at greater risk of mental health issues ( Jellestad *et al*., 2018). Stigmatization, both interpersonally and at a larger social level, is considered to be the leading cause of psychiatric disorders among TW ( Verbeek *et al*., 2020). From a young age, TW often experience bullying and discrimination at school and have been prohibited from expressing their gender identity, such as through wearing feminine uniforms or hairstyles due to the Thai educational policy. Some TW discontinued their education as a result and practiced sex work due to lower educational level and discrimination ( de Lind van Wijngaarden & Fongkaew, 2020).

Additionally, TW can suffer from gender dysphoria (GD) ( Zucker *et al*., 2016). As well as being a consequence of gender incongruity, some psychiatric conditions could mask GD manifestation and interfere with diagnoses of GD. Gender identity development is affected by both biological and psychological factors ( Steensma *et al*., 2013). Psychiatric disorders with biological or psychological origin can also influence identity formation and expression. Moreover, feelings of shame, which inhibit clients’ self-disclosure in order to avoid social discrimination, are more frequently found in Asian cultures compared to Western cultures ( Bedford & Hwang, 2003). Therefore, fear of social rejection can prevent TW from disclosing their genuine gender identity to their peers or even family members ( Huang *et al*., 2020). Providers in TW healthcare could find difficulties in complex psychiatric comorbidities and cultural considerations in diagnoses, treatment planning, gender-affirming therapy, and psychological interventions.

In this study, we portray the clinical experience of three TW with GD and psychiatric comorbidities at our Gender Health Clinic located in Bangkok, Thailand. All these clients were undergoing gender-affirming hormonal therapy (GAHT), which included regular hormonal prescription and pre-procedural psychological counselling. GD was diagnosed by a psychiatrist according to the DSM-5 criteria and psychiatric follow-ups were provided as appropriate. An initial evaluation was conducted to identify those who have contraindication for GAHT, such as active liver disease, hormone-responsive malignancy, or a history of venous thromboembolism. Clients with no contraindications would be prescribed with either transdermal or oral estrogen, and antiandrogen for TW with intact gonads. Clients would be monitored every three months to ensure the safety and efficacy of the treatment. Once their condition is stable, the visit would be every 6-12 months for holistic health surveillance. TW healthcare providers should recognize these patterns so that proper evaluation and holistic care can be delivered to our TW clients.

## Cases

### Cultural considerations and GD 

**TW1** is a 37-year-old Thai engineer from Bangkok. She had been married for four years before visiting our clinic. Brought up in a large Thai-Chinese, multigenerational family, she had carried expectations from her family to continue the family linage. She was sent to a boys’ school and was bought up as a boy. At that time, she reported a sense of maleness and had relationships with women. After her graduation, she worked as an engineer and married her female spouse two years later. Afterwards, she and her wife moved out from her former residence and lived away from her family. She found her authentic femininity following the birth of her son and the experience of becoming a parent for the first time. Independence from her family values gradually revealed masked anxiety as a result of conflict between her genuine gender and traditional Asian social expectations. Fears of discrimination and worrying about the consequences of stigmatization was consciously exhibited and manifested as anxiety symptoms, which GD also contributed to. She ultimately decided to undergo her transition at the age of thirty-five. She has now received GAHT and psychological counselling, which has remarkably improved her distress as well as family problems. Even though her family hoped for a lineal descendent, they also wanted her to be happy and who she truly is.

GD typically manifests very early in childhood ( Zaliznyak *et al*., 2020). Onset GD in TW is most commonly found among adolescents after puberty, and less frequently in adulthood ( Zucker *et al*., 2016). Two crucial points should be mentioned on whether the onset of GD represented being a TW or was additional to this. Those with late-onset GD may prioritize career achievement rather than early transition or have concerns around social discrimination ( Zavlin *et al*., 2019). However, delayed onset of GD has received less academic attention. **TW1** demonstrated prolonged repressed femininity influenced by Asian family values, which expect the continuation of the patriarchal family line ( Berry, 2001). Negative attitudes toward gender minorities are highly prevalent in the Asian context as displayed in the case here. We identified increasing psychological distress in families with lower gender-related support but higher gender-related discrimination ( Fuller & Riggs, 2018; Xie & Peng, 2018). Multidimensional assessment, including family evaluation based on specific cultural belief, should be considered in TW such as our client with adulthood onset GD ( Silveira *et al*., 2016).

### Autism and delayed diagnosis of GD

**TW2** is a 22-year-old TW, studying computer graphic design in Bangkok. She was the only child with a history of being bullied and discriminated against. She was diagnosed with attention deficit/hyperactivity disorder and major depression in childhood. After receiving psychiatric treatment, her symptoms partially improved, but conflicts among her family remained. She frequently had arguments with her mother, who was over-involved in most of her decision-making. Her father was distant and invariably not involved in these disputes. Because of problems at school and with family, she gradually developed more feelings of inadequacy and low self-esteem. It wasn’t until she won a cross-dressing competition at her school festival, that she felt for the first time that her parents were proud of their only son. Later on, she began to dress in feminine fashion, which she found to be her only method of self-soothing, and she was still interested in girls. However, when cross-dressing, she found her male appearance incongruous with her identity, so she began taking oral contraceptive pills to become more feminine. At her age of twenty, her mother was concerned and brought her to our clinic. At the first visit, she appeared in feminine cut shoes and had difficulties in communication with poor eye contact. Because of her communication difficulties, the primary diagnoses were autism spectrum disorder (ASD) and transvestism, with GD as a differential diagnosis. After sessions of counseling and psychoeducation, she was able to describe more about her identity, which was found to be feminine. Her sexual orientation expanded from gynephilia to any gender, and transvestism was replaced with GD three months after her first visit. She then received GAHT which she was satisfied with and had minimal tolerable side effects. She and her family noted the resolution of her distress and improved affect regulation, which lessened her family conflicts.

**TW2** presented an interesting case of GD with co-occurring ASD. The ASD population has greater numbers of gender diversity than the general population ( Glidden *et al*., 2016). Compared to the cisgender population, previous studies consistently found a higher prevalence of neurodevelopmental disorders, including ASD, among transgender people ( Heylens *et al*., 2018; Strauss *et al*., 2021). Specifically, ASD is more common in TW than in transmasculine individuals ( Strang *et al*., 2018). Difficulties in communication and self-understanding regarding their identities and sexual desires make it challenging for providers of transgender healthcare to diagnose GD, therefore more extended periods for evaluation before gender-affirming therapy are required. As presented in our client who has experienced multiple psychosocial problems accompanied by ASD, several psychiatric comorbidities are more common among TW with ASD than those without ASD ( Strauss *et al*., 2021). Clinicians taking care of GD and ASD individuals should be aware of these co-occurrences in order to provide comprehensive mental health surveillance among this population.

### Trans lesbian and borderline personality disorder

**TW3** is a twenty-four-year-old trans lesbian (homosexual TW) living in Bangkok and working as a programmer. After her parents divorced during her infancy, she was brought up by her mother and two elder sisters. Her kindergarten teacher reported her feminine expression to her mother; from then on, she had to suppress her identity to prevent social stigmatization and opposition from family. With little family support, she was forced to engage with entirely male peers, was still interested in women, and became ordained into the Buddhism monkhood as her mother wished. She left her family to go to boarding high school and studied abroad afterwards. During this time, she found herself more comfortable and was able to express her identity freely. However, she then moved back to Thailand, worked as a programmer, and lived alternately with her family and girlfriend. Severe depressive symptoms, including multiple suicidal attempts, gradually arose as a result of relationship problems, discrimination, and her internalized homophobia. She was hospitalized with major depression and borderline personality disorder and referred to our clinic for her previously diagnosed GD. Alongside several psychotropic medications, individual and group psychotherapies, and even electroconvulsive therapy, she has been prescribed GAHT but has reported only a marginal improvement in her conditions.

Borderline personality disorder is a complicated psychiatric disorder presenting affective and relationship instability that always coexists with other mental morbidities ( Paris, 2018). GD and major depression are related in patients with borderline personality and cause emotional regulation difficulties, early life stress and abuse, and identity disturbances along with a higher prevalence of homosexuality ( Carvalho Fernando *et al*., 2014; Frías *et al*., 2016). Unstable self-identity also complicates the diagnosis of GD and requires careful history taking and frequent updates to the patients’ information ( Goldhammer *et al*., 2019). TW with identity problems should be screened for comorbid borderline personality because gender-affirming therapy alone without psychiatric treatments, and vice versa, may result in poorer outcomes ( Smith *et al*., 2019). However, determining the severity of psychopathology is essential to the treatment prognoses presented in **TW3**.

## Conclusion

These case reports demonstrate complex psychiatric comorbidities with GD, which could be underrecognized and untreated. Our cases’ patterns might be common and beneficial in terms of early recognition. However, some limitations including cultural differences should be considered in our study’s generalizability.

In the context of Asian culture and tradition, Thai TW have experienced great internal stigmatization and social discrimination ( de Lind van Wijngaarden & Fongkaew, 2020), which can result in secondary anxiety and delay the timing of identity disclosure and even their own identity recognition. Neurodevelopmental disorders often co-occur and interfere with gender identity development. Communication deficit among the patients could obscure diagnoses of GD and related conditions. Borderline personality disorder often relates to identity disturbance, including gender identity and sexual orientation. TW with borderline personality disorder represent the diversity of sex and gender due to their identity disturbance and highlight the factors that should be considered in treatment. Healthcare providers for TW should be aware of complex psychiatric comorbidities in order to prescribe holistic gender-affirming interventions. Appropriate psychiatric referral is recommended to promote broader physical and mental dimensions of healthcare for all TW.

## Data availability

All data underlying the results are available as part of the article and no additional source data are required.

## Consent

Written informed consent for publication of their clinical details was obtained from the clients.

## Ethical approval

This report was exempted by the Institutional Review Board of the Faculty of Medicine, Chulalongkorn University, Bangkok, Thailand. (COE No.050/2021) Date of exemption: September 28, 2021.

## Author contributions

Sorawit Wainipitapong: Conception and design of the study, Acquisition of data, Analysis and interpretation of data, Drafting the article, Revising it critically for important intellectual content, Final approval of the completed article;

Soravit Burakitpachai: Acquisition of data, Analysis and interpretation of data, Drafting the article;

Thanapob Bumphenkiatikul: Acquisition of data, Analysis and interpretation of data, Drafting the article, Revising it critically for important intellectual content.

## References

[ref1] Bedford O Hwang K-K : Guilt and Shame in Chinese Culture: A Cross-Cultural Framework from the Perspective of Morality and Identity. *J. Theory Soc. Behav.* 2003;33:127–144. 10.1111/1468-5914.00210

[ref2] Berry C : Asian values, family values: Film, video, and lesbian and gay identities. *J. Homosex.* 2001;40(3–4):211–231. 10.1300/J082v40n03_11 11386334

[ref3] Carvalho Fernando S Beblo T Schlosser N : The impact of self-reported childhood trauma on emotion regulation in borderline personality disorder and major depression. *Journal of Trauma & Dissociation: The Official Journal of the International Society for the Study of Dissociation (ISSD).* 2014;15(4):384–401. 10.1080/15299732.2013.863262 24283697

[ref4] Lind van Wijngaarden JW de Fongkaew K : “Being Born like This, I Have No Right to Make Anybody Listen to Me”: Understanding Different Forms of Stigma among Thai Transgender Women Living with HIV in Thailand. *J. Homosex.* 2020;68:2533–2550. 10.1080/00918369.2020.1809892 32841090

[ref5] Frías Á Palma C Farriols N : Sexuality-related issues in borderline personality disorder: A comprehensive review. *Personal. Ment. Health.* 2016;10(3):216–231. 10.1002/pmh.1330 26840032

[ref6] Fuller KA Riggs DW : Family support and discrimination and their relationship to psychological distress and resilience amongst transgender people. *Int. J. Transgenderism* 2018;19(4):379–388. 10.1080/15532739.2018.1500966

[ref7] Glidden D Bouman WP Jones BA : Gender Dysphoria and Autism Spectrum Disorder: A Systematic Review of the Literature. *Sex. Med. Rev.* 2016;4(1):3–14. 10.1016/j.sxmr.2015.10.003 27872002

[ref8] Goldhammer H Crall C Keuroghlian AS : Distinguishing and Addressing Gender Minority Stress and Borderline Personality Symptoms. *Harv. Rev. Psychiatry.* 2019;27(5):317–325. 10.1097/HRP.0000000000000234 31490187

[ref9] Heylens G Aspeslagh L Dierickx J : The Co-occurrence of Gender Dysphoria and Autism Spectrum Disorder in Adults: An Analysis of Cross-Sectional and Clinical Chart Data. *J. Autism Dev. Disord.* 2018;48(6):2217–2223. 10.1007/s10803-018-3480-6 29427119

[ref10] Huang Y-P Wang S-Y Kellett U : Shame, Suffering, and Believing in the Family: The Experiences of Grandmothers of a Grandchild With a Developmental Delay or Disability in the Context of Chinese Culture. *J. Fam. Nurs.* 2020;26(1):52–64. 10.1177/1074840719895264 31910721

[ref11] Jellestad L Jäggi T Corbisiero S : Quality of Life in Transitioned Trans Persons: A Retrospective Cross-Sectional Cohort Study. *Biomed. Res. Int.* 2018;2018:1–10. 10.1155/2018/8684625 29850582 PMC5925023

[ref12] Paris J : Differential Diagnosis of Borderline Personality Disorder. *Psychiatr. Clin. North Am.* 2018;41(4):575–582. 10.1016/j.psc.2018.07.001 30447725

[ref13] Schulman JK Erickson-Schroth L : Mental Health in Sexual Minority and Transgender Women. *Med. Clin. North Am.* 2019;103(4):723–733. 10.1016/j.mcna.2019.02.005 31078203

[ref14] Silveira MT Knobloch F Silva Janovsky CCP : Gender Dysphoria in a 62-Year-Old Genetic Female With Congenital Adrenal Hyperplasia. *Arch. Sex. Behav.* 2016;45(7):1871–1875. 10.1007/s10508-016-0750-2 27270635

[ref15] Smith WB Goldhammer H Keuroghlian AS : Affirming Gender Identity of Patients With Serious Mental Illness. *Psychiatric Services (Washington, D.C.).* 2019;70(1):65–67. 10.1176/appi.ps.201800232 30332928

[ref16] Steensma TD Kreukels BPC Vries ALC de : Gender identity development in adolescence. *Horm. Behav.* 2013;64(2):288–297. 10.1016/j.yhbeh.2013.02.020 23998673

[ref17] Strang JF Meagher H Kenworthy L : Initial Clinical Guidelines for Co-Occurring Autism Spectrum Disorder and Gender Dysphoria or Incongruence in Adolescents. *Journal of Clinical Child and Adolescent Psychology: The Official Journal for the Society of Clinical Child and Adolescent Psychology, American Psychological Association, Division 53.* 2018;47(1):105–115. 10.1080/15374416.2016.1228462 27775428

[ref18] Strauss P Cook A Watson V : Mental health difficulties among trans and gender diverse young people with an autism spectrum disorder (ASD): Findings from Trans Pathways. *J. Psychiatr. Res.* 2021;137:360–367. 10.1016/j.jpsychires.2021.03.005 33761424

[ref19] Verbeek MJA Hommes MA Stutterheim SE : Experiences with stigmatization among transgender individuals after transition: A qualitative study in the Netherlands. *Int. J. Transgender Health.* 2020;21(2):220–233. 10.1080/26895269.2020.1750529 33015671 PMC7430421

[ref20] Xie Y Peng M : Attitudes Toward Homosexuality in China: Exploring the Effects of Religion, Modernizing Factors, and Traditional Culture. *J. Homosex.* 2018;65(13):1758–1787. 10.1080/00918369.2017.1386025 28956732

[ref21] Zaliznyak M Bresee C Garcia MM : Age at First Experience of Gender Dysphoria Among Transgender Adults Seeking Gender-Affirming Surgery. *JAMA Netw. Open.* 2020;3(3):e201236. 10.1001/jamanetworkopen.2020.1236 32176303 PMC7076333

[ref22] Zavlin D Wassersug RJ Chegireddy V : Age-Related Differences for Male-to-Female Transgender Patients Undergoing Gender-Affirming Surgery. *Sex. Med.* 2019;7(1):86–93. 10.1016/j.esxm.2018.11.005 30638830 PMC6377379

[ref23] Zucker KJ Lawrence AA Kreukels BPC : Gender Dysphoria in Adults. *Annu. Rev. Clin. Psychol.* 2016;12:217–247. 10.1146/annurev-clinpsy-021815-093034 26788901

