# Supplementary material for: Complex psychiatric comorbidities in Thai trans women: A case series
Source: F1000Res. 2022 Jan 21;11:74. [Version 1] doi: 10.12688/f1000research.75144.1 (PMC9877471; doi:10.12688/f1000research.75144.1)
Supplement: Peer review history

# Peer review: Reviewer response for version 1

**DOI:** 10.5256/f1000research.78971.r160175

**Published:** 2023-01-25

**Authors:** Gonzalo R Quintana

**Affiliations:** 1 Quintana Zunino, Departamento de Psicología y Filosofía, Facultad de Ciencias Sociales y Jurídicas, Universidad de Tarapacá, Arica, Chile

The article provides a good description of a rarely studied phenomena of psychiatric comorbidity amongst transgender women. They provide valuable evidence on a research area with limited information, where cases analysed from a cultural perspective is highly necessary, and review succinctly, but effectively, the phenomenon of gender dysphoria with relevant citations and authors. The article provides a balanced discussion of each case, especially when considering culture as a determinant factor in each case.

 Their report reads well and fluently. There are minor details highlighted and suggestions below.

 All three cases are adults, between younger and emerging, and middle-age. As pointed out by the authors, GD is most commonly found in adolescence. Therefore, I believe the authors should develop more this aspect of their cases in the conclusions, especially how it factors in the other psychiatric comorbidities. This is a key aspect of their cases that deserves a deeper analysis, especially for future cases.

 Minor comments:

 "Additionally," is not a good word to begin a paragraph. It may be taken out, and perhaps be replaced by the word "also", namely, "TW can ALSO suffer from..."

 Where it says " GD, therefore more..." the comma can very well be changed by a dot.

 Authors should consider enlightening the reader with other studies regarding other psychiatric comorbidities (e.g., mood disorder) with bigger samples of trans people (e.g. Wanta JW, Niforatos JD, 2019; Reisner SL, Biello KB, 2016). ^1^^,^^2^

Are enough details provided of any physical examination and diagnostic tests, treatment given and outcomes?

Yes

Is the background of the cases’ history and progression described in sufficient detail?

Yes

Is sufficient discussion included of the importance of the findings and their relevance to future understanding of disease processes, diagnosis or treatment?

Partly

Is the conclusion balanced and justified on the basis of the findings?

Yes

Reviewer Expertise:

Sexual behavior and function in animals and people.

I confirm that I have read this submission and believe that I have an appropriate level of expertise to confirm that it is of an acceptable scientific standard.

## References

[1] : Mental Health Diagnoses Among Transgender Patients in the Clinical Setting: An All-Payer Electronic Health Record Study. *Transgend Health* .2019;4(1) : 10.1089/trgh.2019.0029 313-315 10.1089/trgh.2019.0029 31701012PMC6830528

[2] : Psychiatric Diagnoses and Comorbidities in a Diverse, Multicity Cohort of Young Transgender Women: Baseline Findings From Project LifeSkills. *JAMA Pediatr* .2016;170(5) : 10.1001/jamapediatrics.2016.0067 481-6 10.1001/jamapediatrics.2016.0067 26999485PMC4882090

# Peer review: Reviewer response for version 1

**DOI:** 10.5256/f1000research.78971.r123259

**Published:** 2022-03-28

**Authors:** Awirut Oon-Arom

**Affiliations:** 1 Department of Psychiatry, Faculty of Medicine, Chiang Mai University, Chiang Mai, Thailand

This article describes interesting complex psychiatric cases of transgender women (TGW) in several aspects. The article demonstrates the complicated neurodevelopment disorder (Autism spectrum disorder) and gender dysphoria. Although both disorders are widely reported as dual-diagnosis or concurrent disorder, the consequence of delayed diagnosis and management were rarely discussed. This article also highlighted the diversity of sexual orientation in TGW that might related to personality components such identity disturbance of borderline personality disorder.

 However, there are a couple of minor comments the authors might wish to consider:

 In terms of mental health outcomes, one of the important mental health is depression and quality of life. There is a report about bullying experience, depression, and quality of life among TGW. Please consider additional discussion citations in this topics. ^1^ ^2^

 Other common health risks among gender minority and borderline personality trait or disorder are substance use and sexual health problems. Increased substance use, across most substances, is found among PD and gender & sexual diverse people. Discussion more on these problems/ different patterns of substance use would widen the perspectives of TW3 and might lead the readers to concern more in psychiatric evaluation among these unique population. ^3^ ^4^

Are enough details provided of any physical examination and diagnostic tests, treatment given and outcomes?

Yes

Is the background of the cases’ history and progression described in sufficient detail?

Yes

Is sufficient discussion included of the importance of the findings and their relevance to future understanding of disease processes, diagnosis or treatment?

Yes

Is the conclusion balanced and justified on the basis of the findings?

Yes

Reviewer Expertise:

psychiatry

I confirm that I have read this submission and believe that I have an appropriate level of expertise to confirm that it is of an acceptable scientific standard.

## References

[1] : Experiences of Being Bullied and the Quality of Life of Transgender Women in Chiang Mai Province, Thailand. *Transgender Health* .2022; 10.1089/trgh.2021.0068 10.1089/trgh.2021.0068 PMC1006678337013090

[2] : Experiences and Psychological Wellbeing Outcomes Associated with Bullying in Treatment-Seeking Transgender and Gender-Diverse Youth. *LGBT Health* .6(5) : 10.1089/lgbt.2018.0179 216-226 10.1089/lgbt.2018.0179 31184969

[3] : Are Borderline Personality Symptoms Associated With Compulsive Sexual Behaviors Among Women in Treatment for Substance Use Disorders? An Exploratory Study. *J Clin Psychol* .72(10) : 10.1002/jclp.22310 1077-87 10.1002/jclp.22310 27059090PMC5030119

[4] : High Burden of Mental Health Problems, Substance Use, Violence, and Related Psychosocial Factors in Transgender, Non-Binary, and Gender Diverse Youth and Young Adults. *Arch Sex Behav* .49(2) : 10.1007/s10508-019-01533-9 645-659 10.1007/s10508-019-01533-9 31485801PMC7018588
